# Computer-aided analysis of quercetin mechanism of overcoming docetaxel resistance in docetaxel-resistant prostate cancer

**DOI:** 10.1186/s43141-023-00498-6

**Published:** 2023-04-26

**Authors:** Victor Omoboyede, Ochapa Ibrahim, Haruna Isiyaku Umar, Grace Ayomide Oke, Olugbenga Samson Onile, Prosper Obed Chukwuemeka

**Affiliations:** 1 Department of Biochemistry, School of Life Sciences (SLS), Federal University of Technology Akure, Akure, P.M.B 704 Nigeria; 2 Computer-Aided Therapeutics Laboratory (CATL), School of Life Sciences (SLS), Federal University of Technology Akure, Akure, P.M.B 704 Nigeria; 3 Computer Aided Therapeutics Discovery and Design (CATDD) Group, School of Life Sciences (SLS), Federal University of Technology Akure, Akure, P.M.B 704 Nigeria; 4 Department of Food Science and Technology, School of Agriculture and Agricultural Technology (SAAT), Federal University of Technology Akure, Akure, P.M.B 704 Nigeria; 5 Biotechnology Programme, Department of Biological Sciences, Elizade University, P.M.B, 002 Ilara-Mokin, Ilara-Mokin, 340271 Nigeria; 6 Department of Biotechnology, School of Life Sciences (SLS), Federal University of Technology Akure, Akure, P.M.B 704 Nigeria

**Keywords:** Complementary therapy, Prostate cancer, Docetaxel, Quercetin, Castration-resistant prostate cancer, Genetic alterations

## Abstract

**Background:**

Prostate cancer (PC) is a silent but potent killer among men. In 2018, PC accounted for more than 350, 000 death cases while more than 1.2 million cases were diagnosed. Docetaxel, a chemotherapeutic drug belonging to the taxane family of drugs, is one of the most potent drugs in combating advanced PC. However, PC cells often evolve resistance against the regimen. Hence, necessitating the search for complementary and alternative therapies. Quercetin, a ubiquitous phytocompound with numerous pharmacological properties, has been reported to reverse docetaxel resistance (DR) in docetaxel-resistant prostate cancer (DRPC). Therefore, this study aimed to explore the mechanism via which quercetin reverses DR in DRPC using an integrative functional network and exploratory cancer genomic data analyses.

**Results:**

The putative targets of quercetin were retrieved from relevant databases, while the differentially expressed genes (DEGs) in docetaxel-resistant prostate cancer (DRPC) were identified by analysing microarray data retrieved from the Gene Expression Omnibus (GEO) database. Subsequently, the protein-protein interaction (PPI) network of the overlapping genes between the DEGs and quercetin targets was retrieved from STRING, while the hub genes, which represent the key interacting genes of the network, were identified using the CytoHubba plug-in of Cytoscape. The hub genes were further subjected to a comprehensive analysis aimed at identifying their contribution to the immune microenvironment and overall survival (OS) of PC patients, while their alterations in PC patients were also revealed. The biological roles played by the hub genes in chemotherapeutic resistance include the positive regulation of developmental process, positive regulation of gene expression, negative regulation of cell death, and epithelial cell differentiation among others.

**Conclusion:**

Further analysis revealed epidermal growth factor receptor (EGFR) as the most pertinent target of quercetin in reversing DR in DRPC, while molecular docking simulation revealed an effective interaction between quercetin and EGFR. Ultimately, this study provides a scientific rationale for the further exploration of quercetin as a combinational therapy with docetaxel.

**Supplementary Information:**

The online version contains supplementary material available at 10.1186/s43141-023-00498-6.

## Background

Globally, the observed surge in mortality rate among men is attributable to several factors, of which various diseases, including cancer, are the vanguard. Alarmingly, PC is the second most common cancer and the second leading cause of cancer-related deaths among men after lung cancer. In 2018, PC accounted for 3.8% of cancer-related deaths in men and more than 1.2 million new cases were diagnosed [1]. Surgery and radiotherapy represent the most potent regimen for surmounting localized PC. However, genomic instability and tumour hypoxia within the tumour microenvironment is commonly linked with relapse and development of advanced PC [2, 3]. Androgen receptor (AR) signalling plays a pivotal role in the development and progression of prostate cancer. Consequently, androgen deprivation therapy (ADT) achieved by castration emerged as the standard therapy for metastatic PC [4, 5]. However, PC cells circumvent ADT and evolve mechanisms to restore androgen signalling. Hence, the progression to castration-resistant prostate (CRPC) cancer [6].

Chemotherapeutic approaches are often employed in combating CRPC. Notably, the advent of docetaxel as a complementary and alternative drug for CRPC treatment was associated with an increased prognosis of CRPC patients [7]. Docetaxel is a member of the class of drugs called taxane. In addition to its ability to restrain cell division, it is also known to induce apoptosis in PC cells and inhibit the transcription of AR [8]. However, CRPC often develops mechanisms of resistance to docetaxel [9]. Hence, necessitating the search for alternative or complementary therapies. Interestingly, several drugs that could reverse docetaxel resistance (DR) have been developed. However, the side effects or ineffectiveness associated with their use have posed a major challenge in their clinical applications [10, 11]. Consequently, natural compounds are been explored as viable alternatives.

Quercetin is a bioflavonoid commonly found in fruits and vegetables. It has been reported to be endowed with a panoply of pharmacological properties including anticancer, anti-inflammatory, antioxidant, and anti-viral properties [12]. The anticancer effects of quercetin are well explored in studies. In a study by Yang et al. [13], it was reported that quercetin suppressed PC xenograft tumour growth via the induction of apoptosis and the inhibition of phosphatidylinositol 3-kinase (PI3K)/Akt signal. The study also reported the anti-angiogenic effect of the compound. Furthermore, it has also been recommended as a viable agent in sensitizing chemotherapy-resistant cancer cells to chemotherapeutics [14]. Interestingly, previous in vitro studies have reported that quercetin increased the sensitivity of DRPC cells to docetaxel by inhibiting the activation of AR among many others [15, 16]. However, the molecular mechanism via which quercetin reverses DR by PC and enhances prognosis in PC patients remains mildly explored.

Accumulating advances in bioinformatics have enabled the effective utilization of the enormous data that are generated during various cancer genomics studies. Consequently, the wrangling of cancer genomic data (CGD) has enabled the discovery of druggable targets and targets that enables cancer drug resistance [17]. By using bioinformatics to wrangle CGD, this study aims to identify the pertinent genes and signalling pathways via which quercetin enhances the response of DRPC cells to docetaxel.

## Methods

### Data collection and processing

The potential targets of quercetin were obtained from targets prediction servers including PharmMapper (http://www.lilab-ecust.cn/pharmmapper/) [18], SwissTargetPrediction (http://www.swisstargetprediction.ch) [19], SEA (https://sea.bkslab.org) [20], and canSAR Black (https://cansarblack.icr.ac.uk/) [21]. Subsequently, the microarray data of DR and docetaxel-sensitive (DS) DU-145 cell lines as well as DR and DS PC3 cell lines were obtained from GSE33455 [22]. GEO2R, an online R programming language-based program for microarray data analysis, was utilized to process the data and identify the differentially expressed genes (DEGs) using the default settings. The relevant DEGs were selected based on *p* < 0.05, |Iog FC|> 1 and the presence of a gene symbol. The potential target genes of quercetin in the DEGs of DRPC were identified using Venny 2.0 [23]. The overlapping genes in the Venn diagram were curated for further analysis.

### Analysis of the PPI network and selection of hub genes

To study the interaction between the overlapping genes, the PPI network of the genes was constructed using STRING-DB v11.5 [24] with the organism set to *homo sapiens* and a medium confidence score of 0.40. Cytoscape v3.9.1 software [25] was utilized to visualize the PPI network. Subsequently, the CytoHubba plug-in [26] was used to identify the hub genes based on the degree of interaction.

### Gene ontology and Kyoto Encyclopaedia genes and genomes pathway enrichment analyses

Functional annotation of the hub genes, including GO and KEGG pathway enrichment analyses, was conducted using shinyGo V 0.76.2 (http://bioinformatics.sdstate.edu/go/) [27]. The biological process, cellular component, and molecular function were the three criteria that were used in the GO analysis under the default setting. Subsequently, the in-built graphing tool of the web server was utilized for the enrichment plots of the significant GO and pathways.

### Analysis of the genetic alterations of hub genes

The genetic alterations of the identified hub genes were analyzed using the cBioPortal (https://www.cbioportal.org/) [28, 29]. The hub genes were submitted as a query and searched in 23 studies on prostate cancer. Subsequently, the study with the highest number of alterations was chosen and subjected to further analysis to determine the type of alterations among the prostate cancer samples. More also, mutual exclusivity analysis was performed to explore the mutual alterations among the hub genes pair. Noteworthy, all analyses were performed at a *p*-value < 0.05.

### Analysis of gene expression of the hub genes

The expression level of the hub genes in prostate cancer was compared with that of normal tissues by utilizing the Gene Expression Profiling Interactive Analysis server (http://gepia.cancer-pku.cn/) [30]. This analysis was performed to validate to determine the difference in the expression of hub genes between PC cells and normal cells based on TCGA and GTEX data. All the parameters of the server were kept default except the p-value cut-off which was set to a threshold of 0.05.

### Survival rate and immune cell infiltration level

The contribution of the expression levels of the hub genes to the overall survival (OS) of PC patients was examined using the Gene Expression Profiling Interactive Analysis server (http://gepia.cancer-pku.cn/) [30]. Furthermore, the Tumour Immune Estimation Resource (TIMER) (https://cistrome.shinyapps.io/timer/) [31] was utilized to analyse the correlation between the hub genes and the tumour immunocytes infiltration levels.

### Molecular docking simulation

Molecular docking was performed to understand the binding interaction of quercetin with EGFR. The crystal structure of EGFR (PDB ID: 4G5J) was retrieved from Protein Data Bank (https://www.rcsb.org/). The Dock Prep module of UCSF Chimera v1.10.2 software [32] was utilized to prepare the retrieved structure. The resulting structure from the Dock Prep was subjected to an energy minimization algorithm using the Swiss-PdbViewer v.4.10 software program [33]. The minimization was performed in vacuo with the GROMOS 43B1 parameter set and without field reaction. Noteworthy, the program automatically identifies missing side chains in protein residues and fixes them. Subsequently, the 3D structure of quercetin was retrieved from PubChem (https://pubchem.ncbi.nlm.nih.gov/) [34]. Using the universal force field (UFF) in PyRx v0.8 [35], the geometry of the compound was energy minimized. The prepared proteins and the fully optimized compounds were the imported PyRx v0.8 software for molecular docking. The docking simulation was performed using the vina module [36], integrated into PyRx v0.8 software. The binding hotspot was defined using the area filled by the cognate ligand of the protein, and a grid box was centred along the x, y, and z axes to allow full coverage of the hotspot residues. Ultimately, the 2D and 3D structures of the docked complex were visualized using ligPlot^+^ v.2.2.5 [37] and PyMOL v.2.5.2 freeware programs respectively.

## Results

### Data collection and processing

**Fig. 1:**
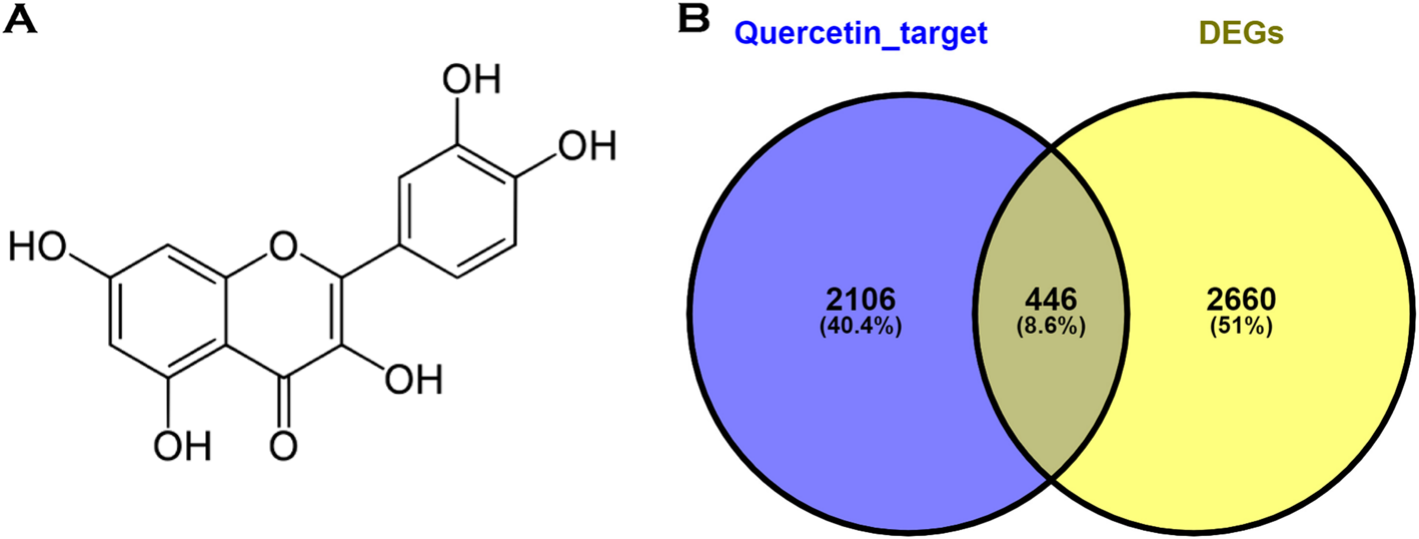
**A** The chemical structure of Quercetin. **B** A Venn diagram showing the overlapping genes between quercetin targets and the DEGs. The left and right circles depict the number of genes that are unique to both quercetin targets and the DEGs respectively while the intersection of the circles depicts the genes that are found in both

A total of 2552 unique targets were retrieved from the utilized target prediction servers. Subsequently, 2339 DEGs consisting of 1151 upregulated genes and 1189 downregulated genes were retrieved from DU-145 cells while 2943 DEGs consisting of 1739 downregulated and 1204 upregulated genes were retrieved from PC3 cells. Ultimately, 3106 unique DEGs were retrieved from the microarray data GSE33455. The Venn diagram server revealed 446 overlapping genes (Fig. 1) which could be considered the target of quercetin in reversing DR in DRPC.

### Analysis of the PPI network and selection of hub genes

**Fig. 2:**
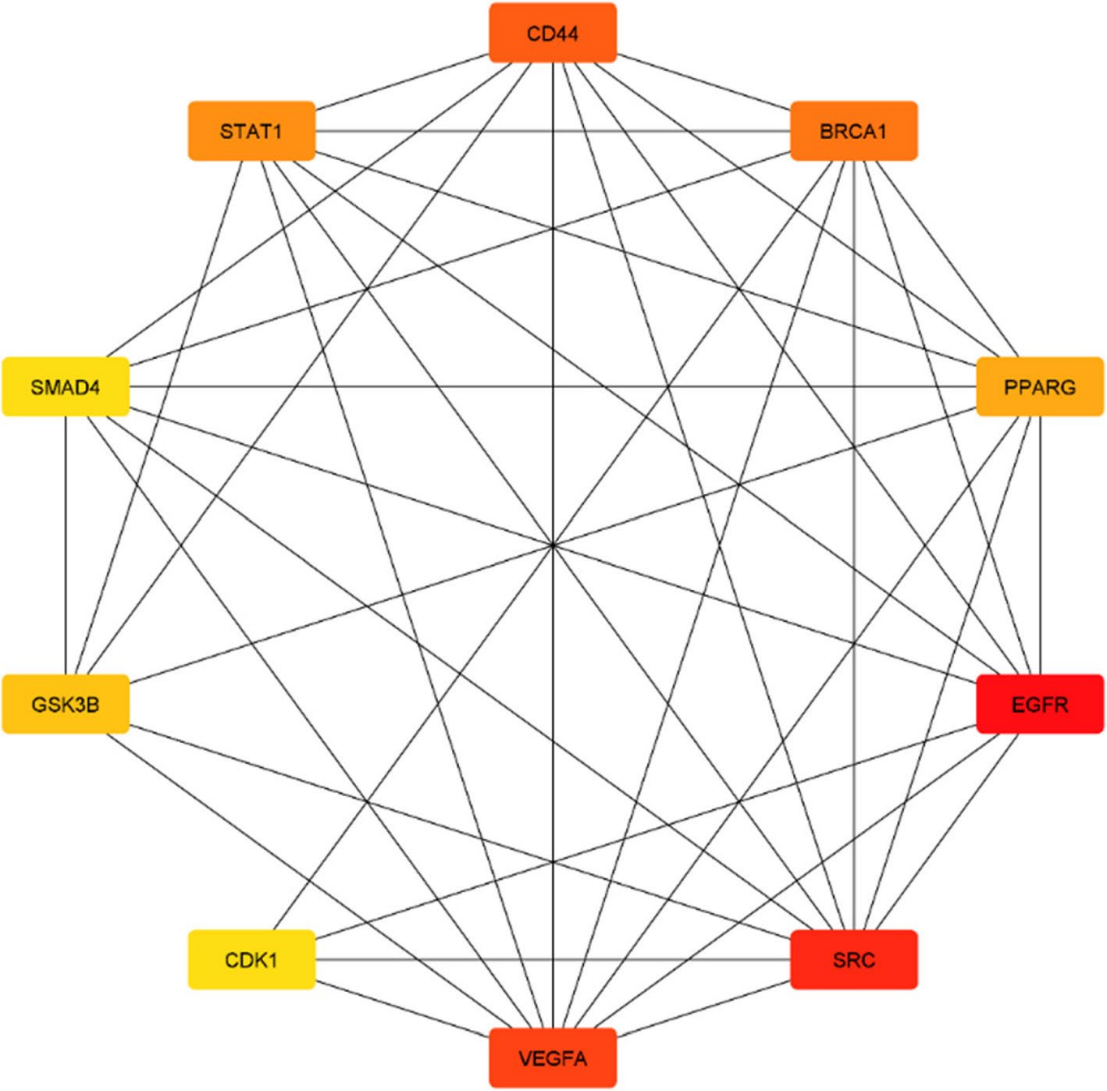
The top 10 interacting targets of the PPI network were retrieved using the CytoHuba plug-in of Cytoscape. The colour represents their degrees of interactions in the initial PPI network, with red representing the top most interacting gene; orange representing the medium most interacting genes; and yellow representing the sparsely interacting genes

To get a better understanding of the interactions between the 446 overlapping targets, STRING-DB v11.5 was utilized to construct a PPI network. The network consists of 424 nodes and 2192 edges with an average local clustering coefficient of 0.39 and a PPI enrichment p-value of < 1.0e-16. Noteworthy, the clustering coefficient measures the connectivity of nodes in a network with highly connected networks having a high value. Furthermore, a Low PPI enrichment p-value is an indication that the nodes and edges are not random but significant. The CytoHubba plugin of Cytoscape was used to select the top 10 hub genes based on the degree score method. The selected hub genes include; VEGFA, SRC, BRCA1, EGFR, PPARG, CD44, STAT1, SMAD4, GSK3B, and CDK1 (Fig. 2). The degrees of connection of the nodes are presented in Table 1.

**Table 1:** The top 10 hub genes and their degree of interactions in the network

Rank	Gene symbol	Degree of interactions
1.	EGFR	102
2.	SRC	95
3.	VEGFA	81
4.	CD44	58
5.	BRCA1	52
6.	STAT1	50
7.	PPARG	46
8.	GSK3B	41
9.	CDK1	40
10.	SMAD4	40

*EGFR* Epidermal growth factor receptor, *SRC* Proto-oncogene tyrosine-protein kinase Src, *VEGFA* Vascular endothelial growth factor A, *CD44* CD44 antigen, *BRCA1* Breast cancer type 1 susceptibility protein, *STAT1* Signal transducer and activator of transcription 1, *PPARG* Peroxisome proliferator-activated receptor gamma, *GSK3B* Glycogen synthase kinase-3 beta, *CDK1* Cyclin-dependent kinase 1, *SMAD4* Mothers against decapentaplegic homolog 4

### GO and KEGG pathway enrichment analyses

**Fig. 3:**
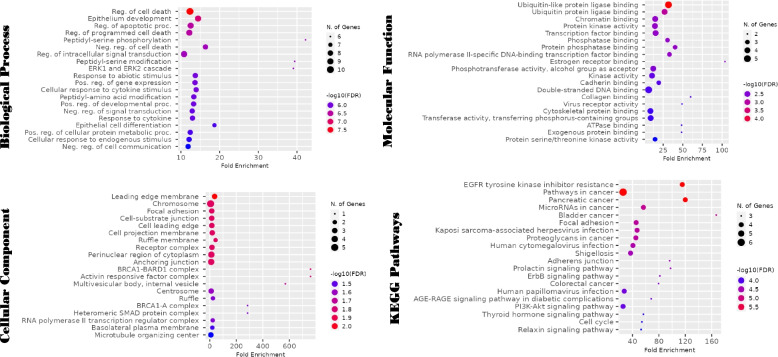
The significant top twenty GO terms and KEGG pathways in which the hub genes are enriched

GO analysis revealed the hub genes were involved in the regulation of several biological processes including the regulation of cell death, epithelium development, and regulation of apoptotic processes. In addition, the hub genes were found to be abundant in the chromosome, perinuclear region of the cytoplasm and anchoring junction. Furthermore, the genes were found to be involved in molecular functions such as ubiquitin-like protein ligase binding, double-stranded DNA binding and transcription factor binding. The KEGG pathways analysis revealed that the genes were enriched in pathways such as pathways in cancer, pancreatic cancer and EGFR tyrosine kinase resistance inhibitor. Figure 3 shows the results of the enrichment plots of the top twenty GO and KEGG pathways.

### Analysis of the genetic alterations of hub genes

**Fig. 4:**
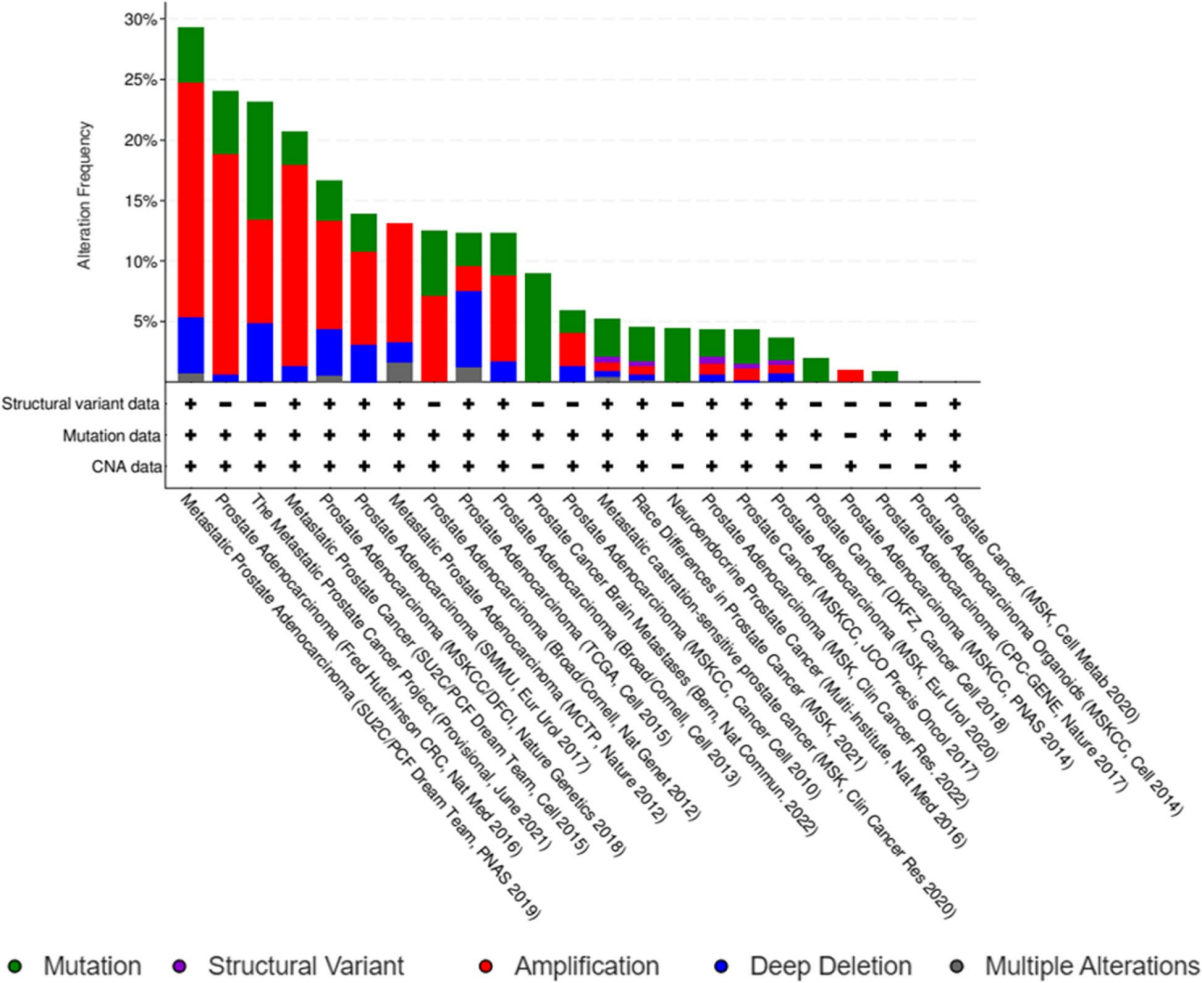
Overview of the genetic alterations of the hub genes derived in this study across 23 PC studies. The different colours present in the bars represent the type of alteration present in each study and the legends are displayed at the lowest part of the plot; the name of each study is displayed are the base of the plot. The “** + **” and “**-**” present at the base of the bar corresponds to the alteration data present in each study, with “** + **” corresponding to present and “**-**” corresponding to absent

**Fig. 5:**
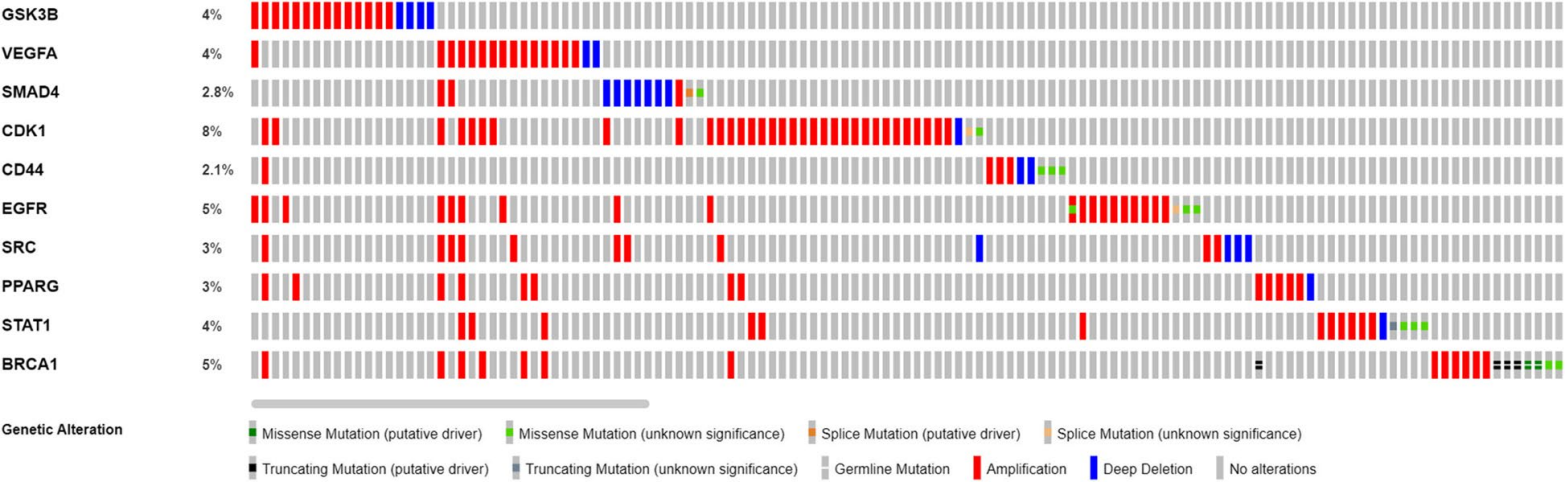
OncoPrint analysis of the genetic alterations of the hub genes in the metastatic prostate adenocarcinoma study (SU2C/PCF Dream Team, PNAS 2019). Each bar corresponds to a patient’s data while the colour of the bar corresponds to the type of genetic alteration present in the patient. The types of genetic alteration the colours correspond to are present in the plot

The genetic alterations of the hub genes across different PC studies were evaluated using cBioPortal. 23 PC studies were analysed for their alteration level while the study with the highest alteration was selected for further analysis (Fig. 4). The study by Abida et al. [38] was selected for further analysis as it contains the highest genetic alterations. The rate of alteration of the hub genes was visualized using OncoPrint. The alteration in the hub genes ranged from 2.1% to 8% in the 429 patient samples studied with amplifications and deep deletion being the most common alteration. The alteration of each hub gene is CD44 (2.1%), SMAD4(2.8%), PPARG (3%), SRC (3%), VEGFA (4%), STAT1 (4%), GSK3B (4%), BRCA1 (5%), EGFR (5%), and CDK1 (8%) (Fig. 5).

The mutual exclusivity test revealed that diverse pairs of genes exhibited significant mutational co-occurrence (Table 2). Analysis of the pathways that are disrupted by the genetic alteration of the hub genes revealed RKT-RAS, WNT, and TGF-Beta pathways as the disrupted pathways. EGFR was present in RKT-RAS, while GSK3B and SMAD4 were found in WNT and TGF-Beta respectively (Supplementary Fig. 1). Interestingly, these pathways are critical for PC development and progression.

**Table 2:** The results of the mutual exclusivity analysis of the hub genes

A	B	Log2 odds ratio	q-value	Tendency
BRCA1	PPARG	> 3	< 0.001	Co-occurrence
SRC	SMAD4	> 3	< 0.004	Co-occurrence
SRC	EGFR	> 3	< 0.004	Co-occurrence
VEGFA	BRCA1	> 3	< 0.007	Co-occurrence
VEGFA	EGFR	> 3	< 0.007	Co-occurrence
VEGFA	SRC	> 3	< 0.008	Co-occurrence
VEGFA	PPARG	> 3	< 0.008	Co-occurrence
SRC	CDK1	2.694	0.022	Co-occurrence
CDK1	PPARG	2.694	0.022	Co-occurrence
SRC	PPARG	> 3	0.033	Co-occurrence
VEGFA	CDK1	2.268	0.047	Co-occurrence

### Analysis of gene expression of the hub genes

**Fig. 6:**
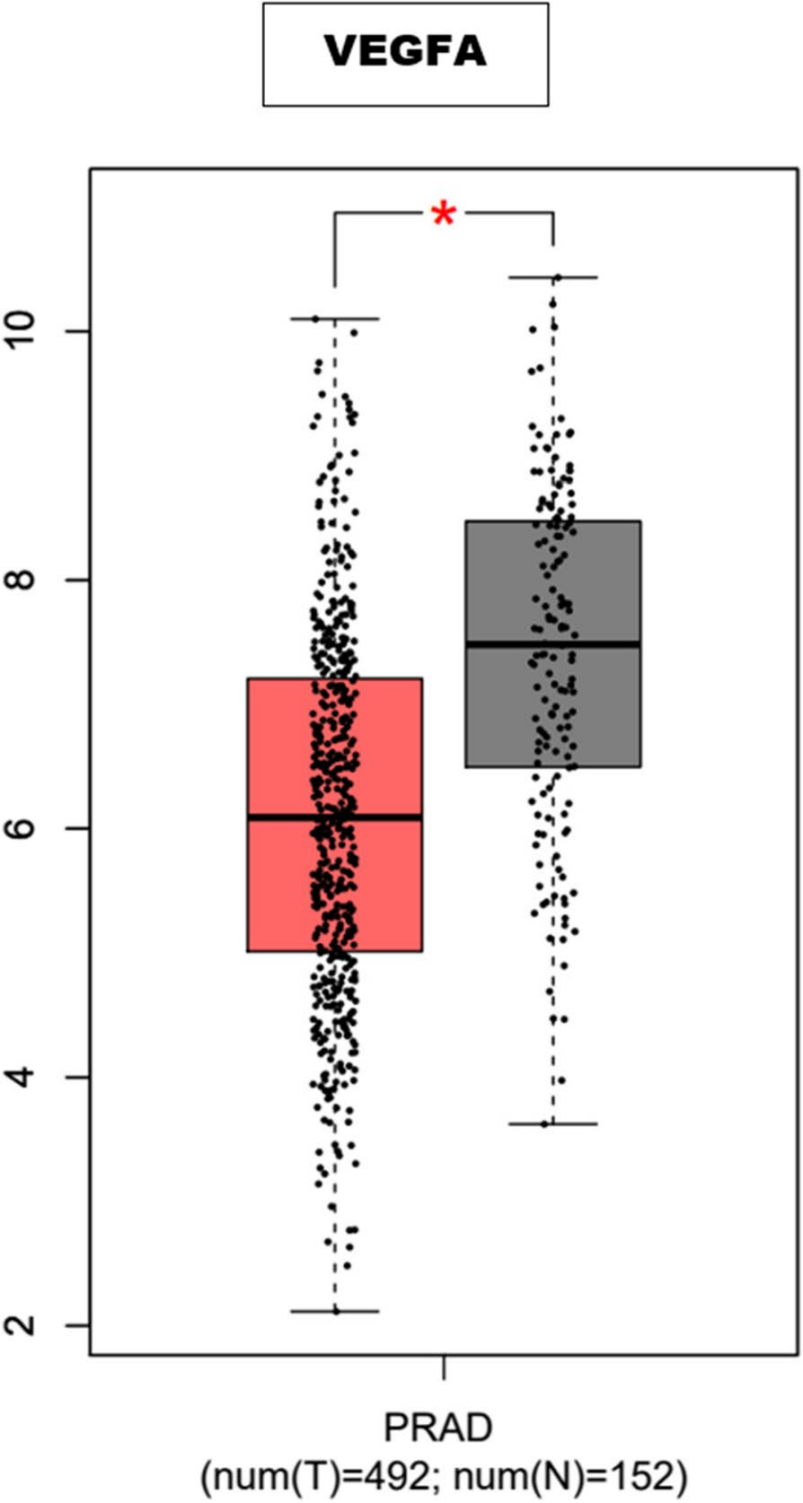
Validation of VEGFA expression in PC cells and normal cells using the TCGA and GTEX data in the GEPIA server. The symbol ∗ symbolizes *p* < 0.05

The mRNA level of hub gene VEGFA was found to be significantly higher in patients with prostate cancer than in normal prostate tissue (Fig. 6). However, the difference between the mRNA levels of EGFR, CDK1, SMAD4, CD44, GSK3B, SRC, PPARG, STAT1 and BRCA1 in prostate cancer and normal tissue were not statistically significant (Supplementary Fig. 2). This could be a result of the amount of data available for PC studies on the webserver or the p-value cut-off that was used in this study.

### Survival rate and immune cell infiltration level

**Fig. 7:**
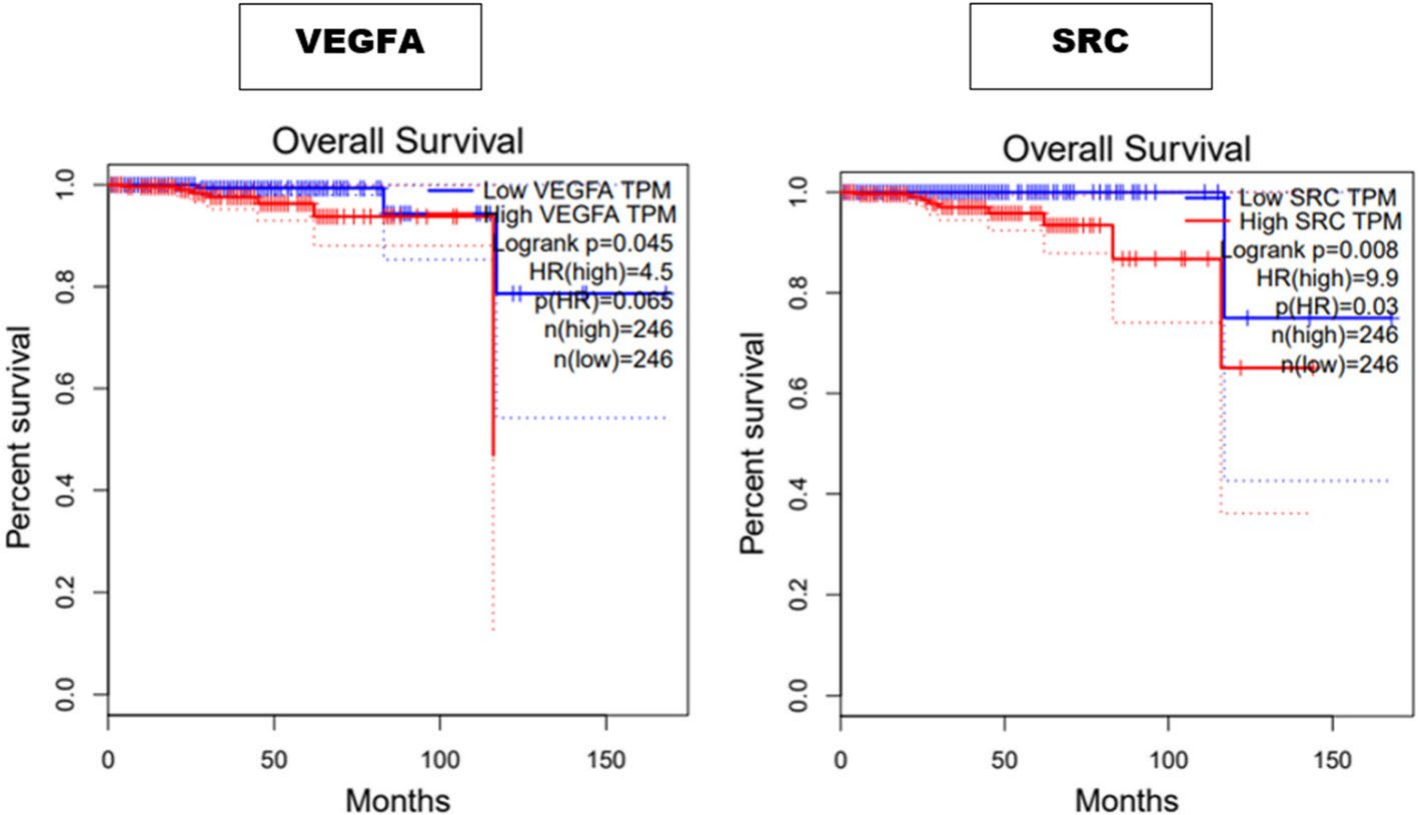
The contribution of the expression levels of VEGFA and SRC to the OS of PC patients based on TCGA data

To ascertain how clinically relevant the expression levels of our hub genes are, we investigated whether the expression levels are related to the OS of PC patients. Low expression levels of VEGFA and SRC were significantly associated with better OS at *P* < 0.05 (Fig. 7).

**Fig. 8:**
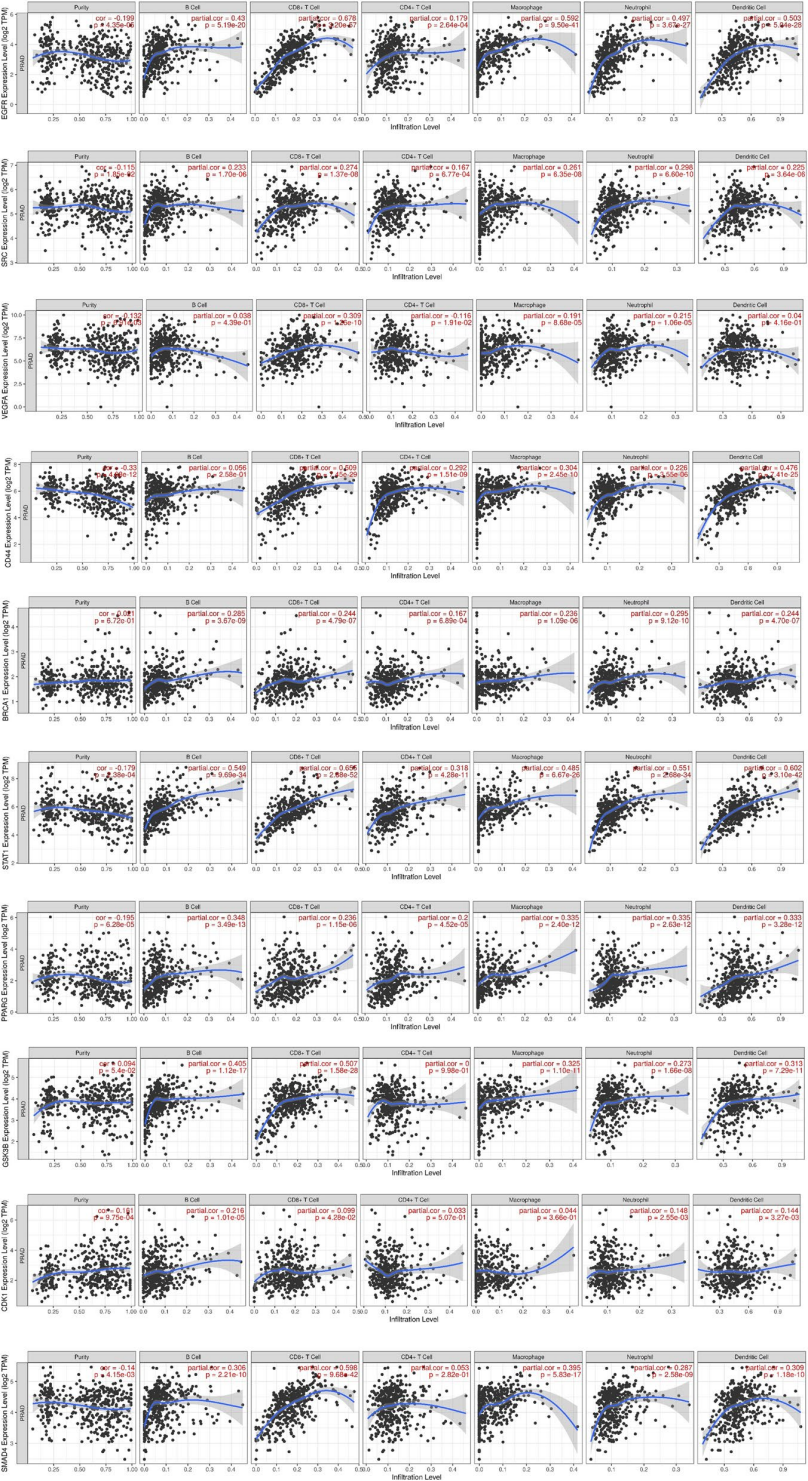
The correlation between the expression levels of the hub genes and the infiltration levels of B cells, CD8 + T cells, CD4 + T cells, macrophages, neutrophils, and dendritic cells

However, other results were not statistically significant (Supplementary Fig. 3). Furthermore, we analysed the relationship between hub genes expression levels and immunocyte infiltration to comprehend the function of the immune microenvironment in the development and prognosis of patients with prostate cancer. The expression levels of EGFR, SRC, VEGFA, CD44, PPARG, SMAD4, and STAT1 were negatively correlated with PC purity but positively correlated with immunocytes infiltration levels. Conversely, the expression levels of BRCA1, CDK1, and GSK3B were found to be positively correlated with both PC purity and immunocyte infiltration level (Fig. 8).

### Molecular docking simulation

**Fig. 9:**
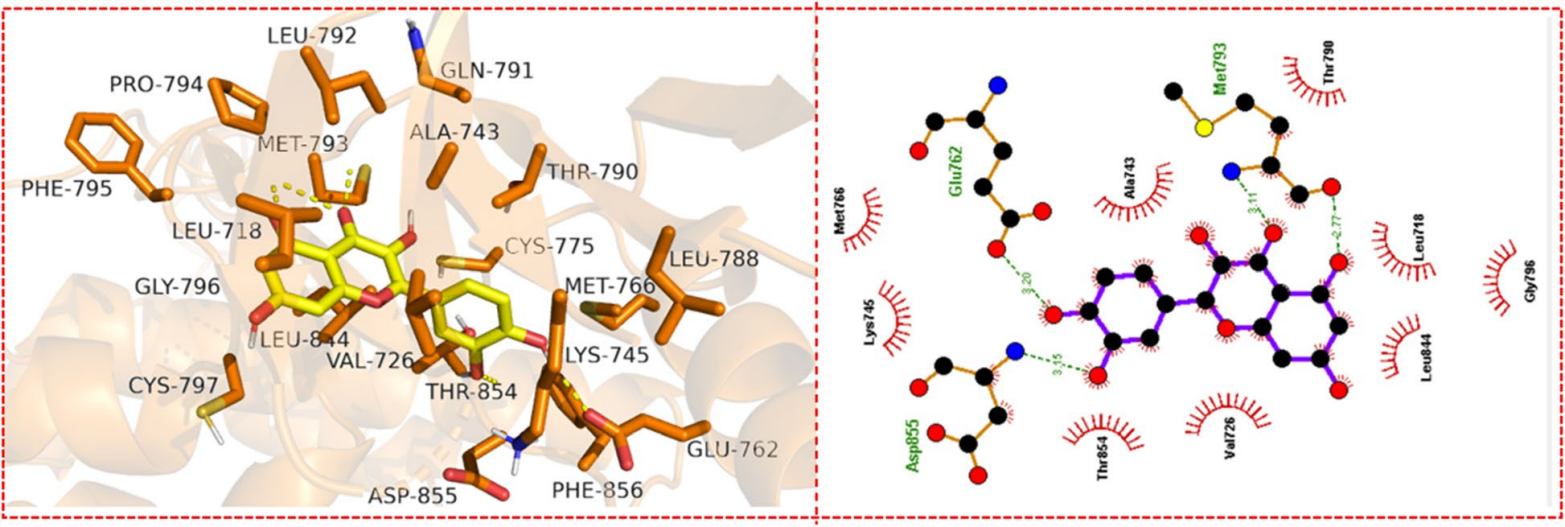
2D and 3D interactions of EGFR-quercetin complex. The left part of the figure depicts the 3D interaction while the right part depicts the 2D interaction

The binding energy of quercetin with EGFR was -8.7 kcal/mol and was found to be lesser compared to that of the native ligand -8.397 kcal/mol. Noteworthy, lower binding energy corresponds to a better affinity. Analysis of the interactions between the docked complex revealed that quercetin formed hydrogen bonds with the protein via GLU762, MET 793, and ASP855. It also formed hydrophobic interactions with LEU718, VAL726, ALA743, LYS745, GLU762, MET766, THR790, MET793, GLY796, LEU844, THR854, and ASP855. The 2D and 3D representations of the interactions are depicted in Fig. 9.

## Discussion

PC is one of the worst types of cancer among men due to its high morbidity and mortality. The use of chemotherapeutics such as docetaxel in its management has gained traction over the years due to its effectiveness. However, the cases of resistance have been a major hurdle in their utilization. While studies have confirmed the potential of quercetin to increase sensitivity to docetaxel, the probable mechanism via which it does is yet to be revealed on a comprehensive level. Consequently, we utilised transcriptome analysis to identify the DEGs that could potentially mediate PC cells’ resistance to docetaxel. Subsequently, the identification of genes that are common to the DEGs and quercetin led to the identification of the group of genes via which quercetin could potentially exert its therapeutic effects. Further analyses to delineate the interactions between the sets of genes via PPI network construction led to the identification of genes that can be considered the core targets of quercetin in DRPC. These sets of genes namely EGFR, SRC, VEGFA, CD44, BRCA1, STAT1, PPARG, GSK3B, CDK1, and SMAD4 have been reported to play numerous roles in tumorigenesis and tumour progression. Analysis of the biological processes mediated by the core targets revealed processes including the regulation of epithelium development, epithelial cell differentiation, negative regulation of cell death, and positive regulation of cellular protein metabolic processes. The pathways in which these core targets were enriched included EGFR tyrosine kinase inhibitor resistance, PI3K-Akt signalling pathway, ErbB signalling pathway, as well as pathways in cancer among others. Further analysis of the PPI network revealed EGFR as the topmost gene that interacted with other proteins present in the network. The alterations of the components of certain pathways including the PI3K/Akt pathway have been reported in most metastatic PC [39]. Consequently, the levels and types of alterations of the hub genes in PC patients were examined. Interestingly, amplification was found to be the major common alteration in most PC patients. Specifically, all the hub genes including key signalling molecules such as CD44, VEGFA, EGFR, SRC, and SMAD4 among others were found to be amplified in PC patients. Notably, CD44 is the most common cancer stem cell receptor, its overexpression has been implicated in cancer chemoresistance, tumour relapse, and metastasis [40]. In this study, amplification was found to be the most significant CD44 genetic alteration. Interestingly, the overexpression of CD44 has been associated with poor prognosis, due to its ability to lower the cytotoxic capacity of chemotherapeutic drugs [41]. Hence, CD44 is a target for the reversal of resistance to chemotherapeutics in PC. Interestingly, a study by Wang et al. [42] reported that quercetin enhanced the therapeutic effects of docetaxel in PC cells by targeting CD44 proteins among many others. In line with this study, the inhibition of CD44 by quercetin is anticipated to reverse DR in DRPC. Similarly, the overexpression of VEGF to which VEGFA belongs has been reported to stimulate the migration of neutrophils and prevent the maturation of dendritic cells, hence, leading to the inactivation of CD8 + T-cells [43, 44]. Furthermore, studies have reported that VEGF also contributes to immunosuppressive TME and CD8 + T-cells depletion by increasing the expression of PD-1 on CD8 + T-cells and CD4 + T-cells [45]. As depicted in Fig. 7, low expression of VEGFA corresponds to better OS in PC patients. Hence, the exertion of quercetin’s reversal effect in DRPC is suspected to be associated with its ability to inhibit VEGFA [46, 47].

It is also worth noting that the expression of EGFR, which was found to be the most important of all the hub genes, has been reported to be induced by docetaxel and this was found to attenuate its therapeutic effects [48]. Furthermore, the level of expression of EGFR was found to correspond to the magnitude of DR. The treatment of DRPC cells with gefitinib, an EGFR inhibitor, increased sensitivity to docetaxel [48]. Hence, the inhibition of EGFR by quercetin can be said to be the most probable mechanism via which quercetin enhances sensitivity to docetaxel. EGFR-mediated expression of ABCB1 in PC cells via an Akt-dependent pathway is also one of the most widely reported mechanisms for DR [48, 49]. Therefore, the inhibition of EGFR can lead to the trigger of a cascade that results in the modulation of ABCB1-mediated DR. Similarly, Zhou et al*.* reported that endothelial cells, through the secretion of fibroblast growth factor (FGF2) attenuate the sensitivity of PC cells to docetaxel by the activation of the Akt/mTOR signalling pathway [50]. Noteworthy, this pathway, which is one of the pathways in which the core targets are enriched, is activated by the phosphorylation of PI3K, which in turn activates Akt, ultimately regulating several downstream molecules including mTOR. In addition to studies demonstrating the pivotal role of EGFR in DRPC, the overexpression of EGFR has been reportedly associated with hormone-refractory behaviour in PC [51]. Therefore, the results of this study reveal that the inhibition of EGFR by quercetin can lead to the blockage of the cascade triggered by EGFR and could ultimately lead to the reversal of DR.

Molecular docking was employed to study the interactions of quercetin with EGFR and its binding affinity. The affinity of quercetin for EGFR was quite high as revealed by the docking score. Interestingly, quercetin interacted with many of the active site amino acid residues of EGFR via four hydrogen bonds and hydrophobic bonds with other residues (Fig. 7). These interactions could potentially be responsible for the modulation of EGFR by quercetin. Hence, the circumventing of DR in DRPC.

## Conclusion

Summarily, this study applied an integrative computational approach to unravel the targets and action mechanisms via which quercetin reverses DR in DRPC. The results of our analysis revealed the core targets via which quercetin reverses DR, while the contribution of the identified targets' expression to OS, and immunocyte infiltration levels was also examined. Furthermore, the various dysregulated pathways which enhance DR in DRPC but could be restored by the therapeutic action of quercetin were identified. Conclusively, the findings of this study revealed EGFR as the most important target of quercetin in DRPC and further establish a scientific rationale for the exploration of quercetin in PC, especially as a combinational therapy with docetaxel in the treatment of DRPC. Nevertheless, the results of this study should be subjected to further in silico, in vitro, and in vivo studies.

## Supplementary Information

**Additional file 1:** **Supplementary figure 1.** The pathways related to genetic alterations predicted by CBioPortal showing that the alterations of EGFR, GSK3B, and SMAD4 dysregulated cell survival, translation, proliferation, and stem/progenitor phenotype. **Supplementary figure 2.** Validation of the hub genes expression in PC and normal cells using the TCGA and GTEX data in GEPIA server. The data depicted by this figure are statistically insignificant. **Supplementary figure 3.** The contribution of the expression levels of the hub genes to the OS of samples of PC patients based on TCGA data.

## Data Availability

All data used in this study are freely available on public databases. The database are; Gene Expression Omnibus (GEO) database (https://www.ncbi.nlm.nih.gov/geo/geo2r/?acc=GSE33455) and CBioPortal (https://www.cbioportal.org/) under the prostate cancer studies. The retrieval processes are clearly stated in the methodology of the manuscript.

## Abbreviations

PC: Prostate cancer
DR: Docetaxel resistance
DRPC: Docetaxel-resistant prostate cancer
PPI: Protein-protein interactions
AR: Androgen receptor
ADT: Androgen deprivation therapy
CRPC: Castration-resistant prostate cancer
DEG: Differentially expressed genes
GO: Gene ontology
KEGG: Kyoto encyclopaedia of genes and genomes
OS: Overall survival
PDB: Protein data bank
TME: Tumour microenvironment
