# Supplementary material for: Computer-aided analysis of quercetin mechanism of overcoming docetaxel resistance in docetaxel-resistant prostate cancer
Source: J Genet Eng Biotechnol. 2023 Apr 26;21:47. doi: 10.1186/s43141-023-00498-6 (PMC10133427; doi:10.1186/s43141-023-00498-6)
Supplement: Supplementary file 1 — Additional file 1: Supplementary figure 1. The pathways related to genetic alterations predicted by CBioPortal showing that the alterations of EGFR, GSK3B, and SMAD4 dysregulated cell survival, translation, proliferation, and stem/progenitor phenotype. Supplementary figure 2. Validation of the hub genes expression in PC and normal cells using the TCGA and GTEX data in GEPIA server. The data depicted by this figure are statistically insignificant. Supplementary figure 3. The contribution of the expression levels of the hub genes to the OS of samples of PC patients based on TCGA data. [file 43141_2023_498_MOESM1_ESM.docx]

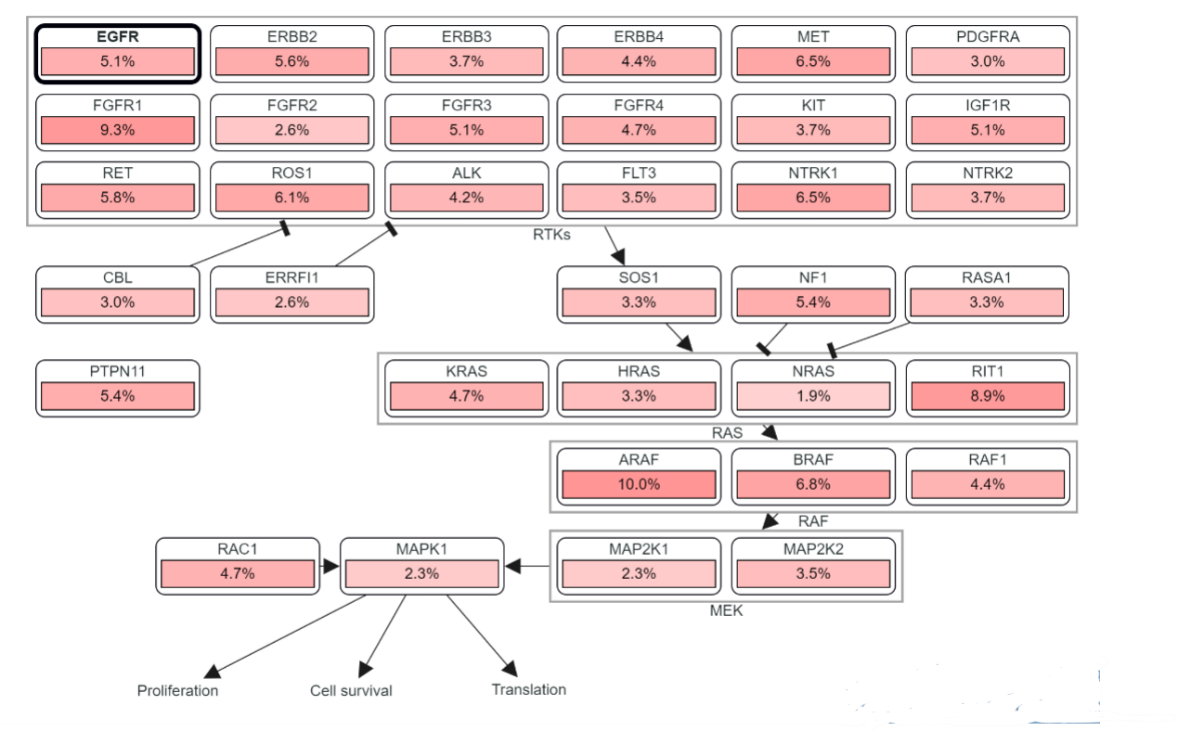

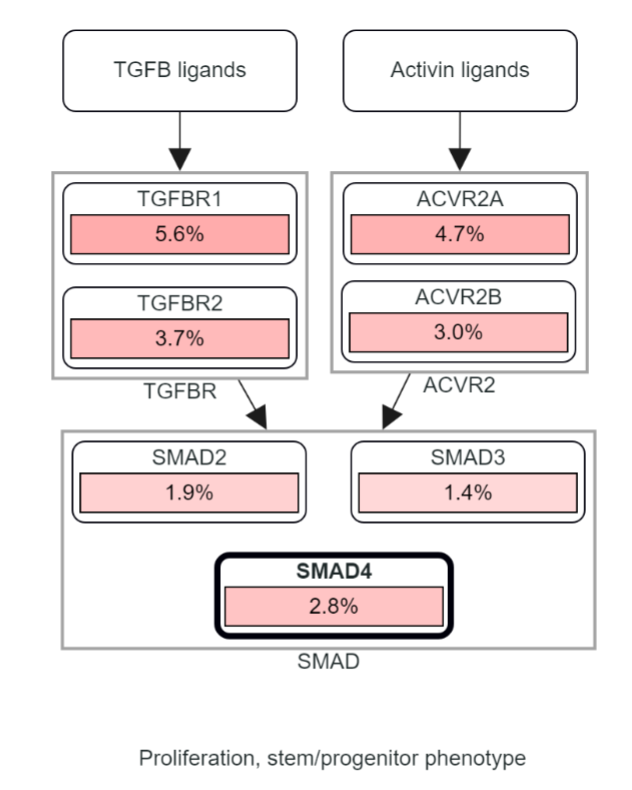


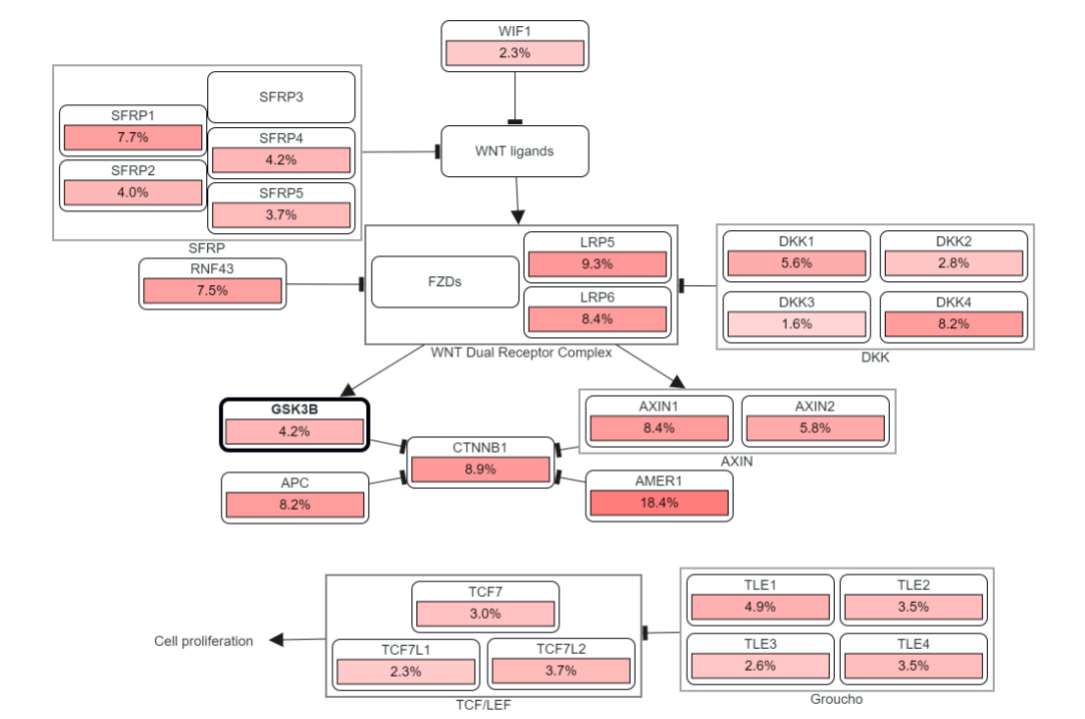


Supplementary figure 1. The pathways related to genetic alterations predicted by CBioPortal showing that the alterations of EGFR, GSK3B, and SMAD4 dysregulated cell survival, translation, proliferation, and stem/progenitor phenotype.


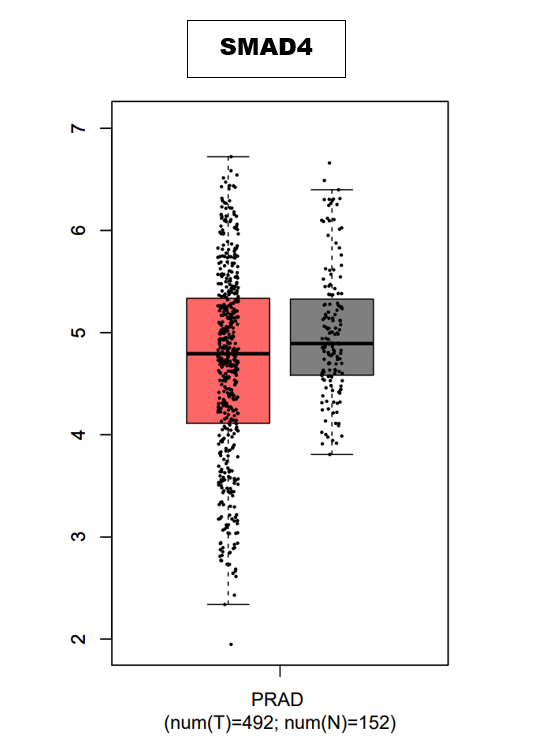

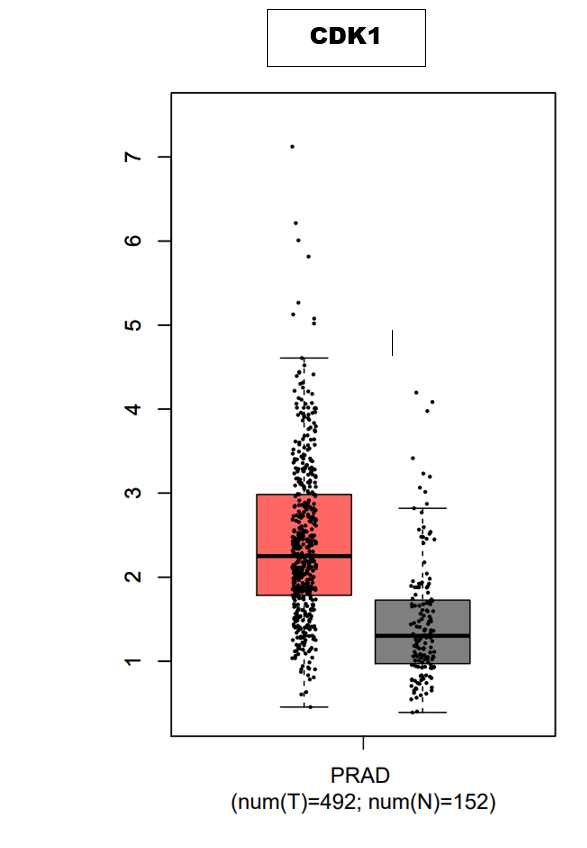

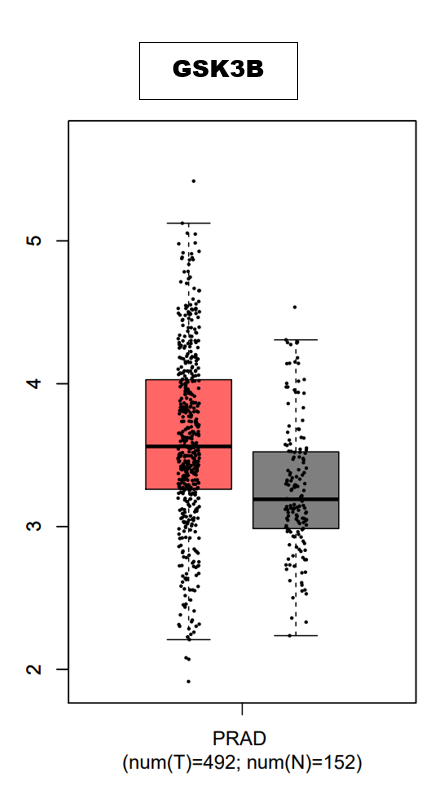


**Continued**


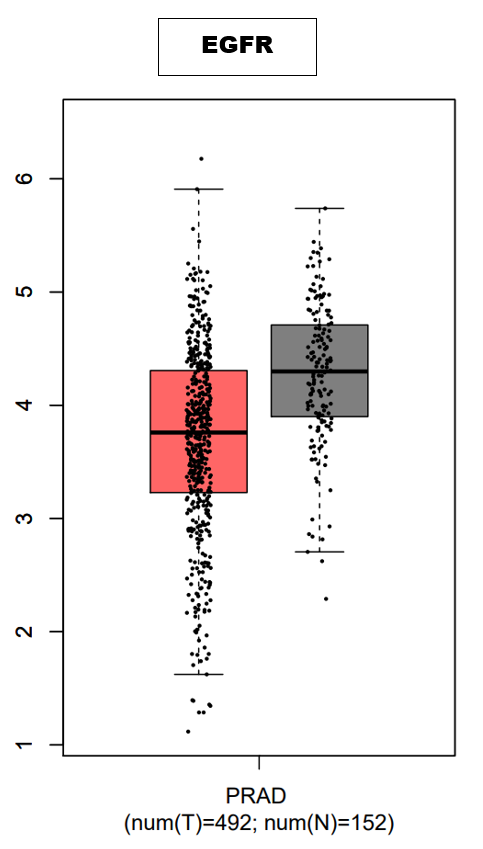

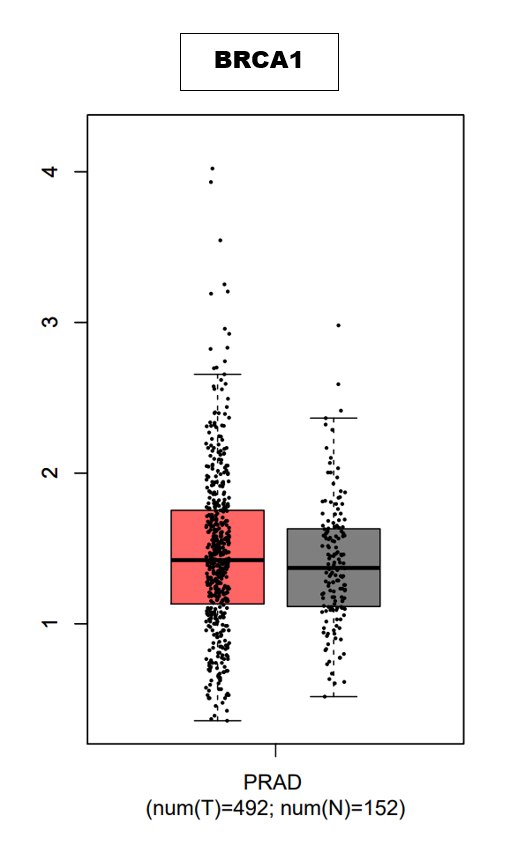

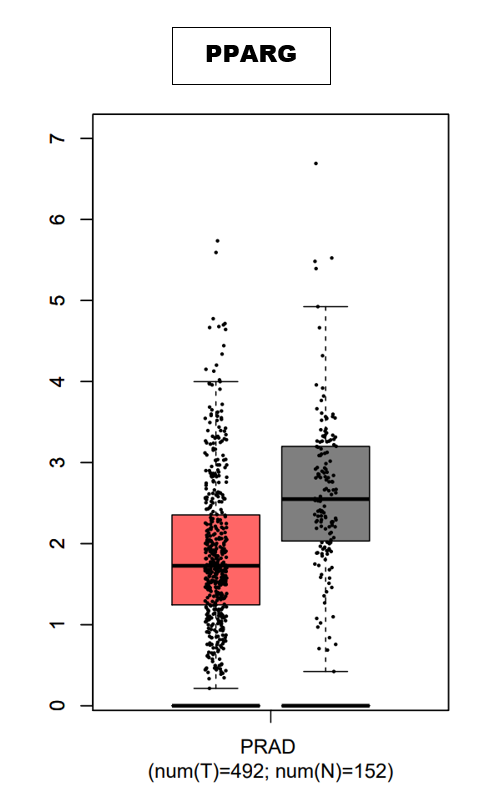


**Continued**


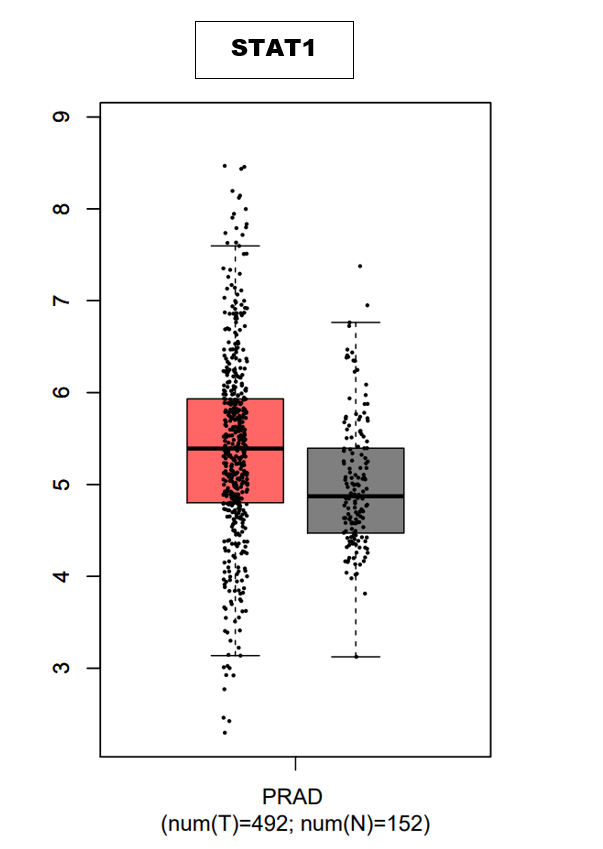

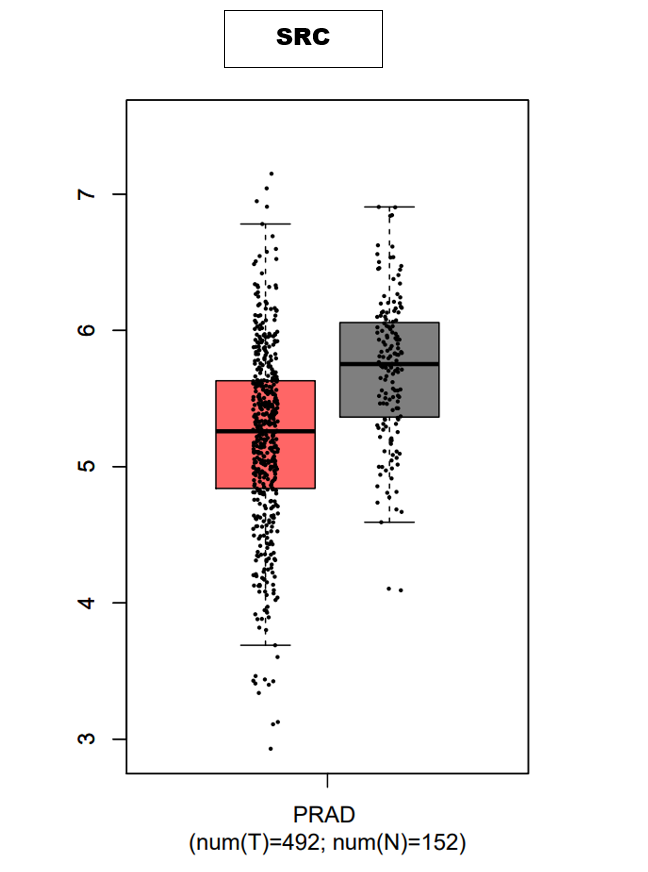

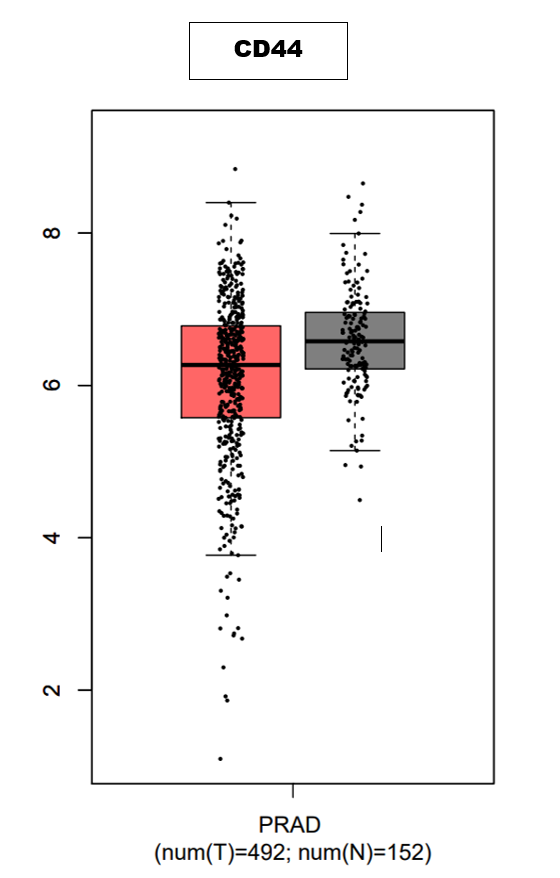


Supplementary figure 2. Validation of the hub genes expression in PC and normal cells using the TCGA and GTEX data in GEPIA server. The data depicted by this figure are statistically insignificant.


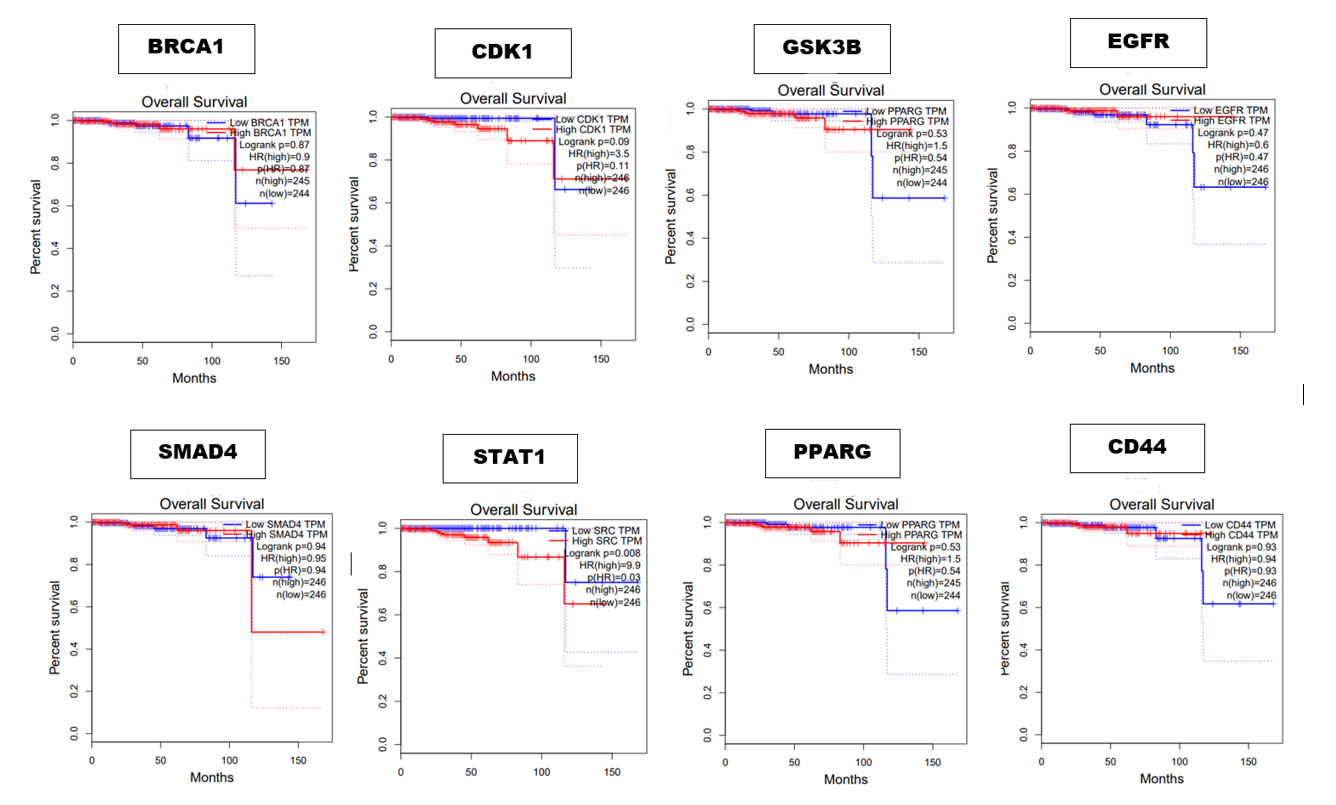


Supplementary figure 3. The contribution of the expression levels of the hub genes to the OS of samples of PC patients based on TCGA data.
